# Gut dysbiosis and immune dysfunction induced by chronic cola replacement of water in rats: not just a sugar problem

**DOI:** 10.3389/fnut.2025.1707842

**Published:** 2026-02-04

**Authors:** Huijie Gao, Wanru Li, Xin Wang, Yan Ren, Xiao Li, Chao Liu

**Affiliations:** College of Pharmacy, Jining Medical University, Rizhao, Shandong, China

**Keywords:** cola beverages, gut dysbiosis, immune dysfunction, renal function, sugar-free vs. sugar-sweetened

## Abstract

**Objective:**

Excessive sugar-sweetened beverage consumption like cola is a worldwide public health problem. However, the effects and mechanisms of replacing water with cola (either as sugar-free cola or sugar-sweetened cola) on intestinal microbiota and overall health remain unknown, prompting this investigation.

**Methods:**

To address this, we created a new experimental model in which cola completely replaced drinking water. Twenty-four Sprague-Dawley rats were randomly divided into three groups (*n* = 8): water, cola (sugar-sweetened), diet cola, for an 8-week intervention. Body weight, body length, BMI, organ indices, hematological, and serum biochemical parameters, and gut microbiota (16S rRNA gene sequencing) were determined.

**Results:**

Both colas resulted in immune suppression (lower thymus index and leukopenia) and decreased total protein. The cola group showed renal stress [higher blood urea nitrogen, (BUN)] and a significant increase in spleen index. The diet cola induced a significant transaminases elevation compared to the cola group. Both colas significantly altered intestinal microbiota structure, including changes in diversity and abundance (e.g., shifts in *Firmicutes* and *Bacteroidota* proportions and in the abundance of *Ligilactobacillus* and *Lactobacillus*). Co-abundance network showed complicated relationships, mostly involving *Lactobacillus, Romboutsia*, and other taxa. Furthermore, *Bacteroidota* and unclassified *Lactobacillus* were significantly correlated with immune organ indices (thymus, spleen) and BUN.

**Conclusion:**

Using an innovative model of complete water replacement, our study demonstrates that long-term cola consumption-whether or not sugar containing-has significant perturbative effects on gut microbiota and impairs immune and renal function. These results should warn of the impacts of regular cola intake and the need for further scrutiny of artificial sweeteners.

## 1 Introduction

In recent years, the global prevalence of metabolic diseases such as obesity and diabetes has drawn significant attention to dietary health issues. High-sugar diets have become a research focus due to their strong association with chronic diseases (1). The World Health Organization (WHO) recommends that daily free sugar intake should be less than 10% of total energy intake. However, overconsumption of sugar-sweetened beverages remains prevalent (2).

Sugar-sweetened beverages, exemplified by cola, typically contain high levels of sucrose or high-fructose corn syrup, which pose health risks. High fructose intake has been directly linked to liver fat accumulation and insulin resistance, and sugar-sweetened beverage consumption is positively associated with the risk of type II diabetes mellitus (3, 4). Consequently, reducing the consumption of sugar-sweetened drinks has become a global public health priority. To reduce sugar intake, sugar-free beverages containing artificial sweeteners such as aspartame have gained popularity as alternatives. Aspartame, one of the most commonly used artificial sweeteners, is metabolized in the body to phenylalanine, aspartic acid, and methanol. The European Food Safety Authority considers it acceptable for use within the established Acceptable Daily Intake, intake of 40 mg/kg body weight (5). However, the long-term safety of these sugar-free beverages remains controversial in light of emerging evidence which indicates that artificial sweeteners may have a deleterious effect on the health of gut microbiota, reducing the amount of good bacteria, increasing the amount of pathogenic bacteria, and disturbing the metabolic pathways related to them (6).

Studies have shown that sugar-sweetened beverages may induce metabolic disorders by altering the intestinal microbiota. However, some experiments have suggested that high-sugar and high-fat diets may not always produce significant effects on intestinal microbiota or health under certain conditions (7–12). This apparent discrepancy may be explained by the contribution of gut dysbiosis to complex systemic pathophysiology, affecting not only local metabolism but also immune and neuroendocrine communication via the gut–brain axis (13). For instance, short-chain fatty acids (SCFAs) have been shown to modulate the host inflammatory response through activation of G-protein-coupled receptors (e.g., GPR43/41), potentially buffering against the effects of dietary perturbations (14). Additionally, it may also be related to the neutralization effect of the components, for example, caffeine has been shown to significantly reduce hepatic steatosis and concomitantly increase autophagy and lipid uptake in lysosomes in mice fed a high-fat diet (15). However, a change in gut microbiota composition that high sugar + high fat diet prompt could also lead to a decrease in SCFAs producing bacteria, attenuating such signaling and yielding a setting conducive to a state of dysregulation at the systemic immune levels (16). Taking into account the complex interactions mentioned above, the specific effects of cola (sugar-free vs. sugar-sweetened) on the gut microbiota and overall health remain unclear, which is precisely the purpose of our research.

16S rRNA sequencing is widely used in gut, environmental and clinical microbiome studies due to its culture-independent nature, high sensitivity, and cost-effectiveness (17). This technique has become an important tool in diet-microbiota interaction studies, enabling analysis of gut microbiota diversity (18). In the present study, we focused on the specific intervention of substituting water with cola to analyze its effects on rat intestinal microbiota and overall health using 16S rRNA sequencing technology. By comparing the differences in gut microbiota between sugar-sweetened and sugar-free colas, we aim to provide complementary evidence on the health risks associated with sugar-sweetened beverages and artificially sweetened “sugar-free” beverages, laying the groundwork for subsequent mechanistic studies (e.g., metabolomics or host-microbiota interactions).

Our work is prompted by two key unanswered questions. One has to do with the long-term health effects, especially on immune and kidney function, of substituting all water intake with cola, and the other with how consumption of sugar-sweetened vs. sugar-free cola affects these outcomes. In the current study we employ a novel exposure model whereby cola substitutes completely for drinking water. This clinically relevant design permits addressing both questions simultaneously. Our model provides critical evidence for dissecting the distinct risks posed by sugars vs. artificial sweeteners in a ubiquitous beverage.

## 2 Materials and methods

### 2.1 Grouping of animals for the experiment

Twenty four Sprague-Dawley (SD) rats (half male and half female, 6–8 weeks old; 100 ± 20 g body mass) were selected for this experiment; they were housed in an experimental animal facility at a temperature of (25 ± 2) °C with a humidity of (50 ± 10) %. All of the rats were fed a standard laboratory rodent chow *ad libitum* throughout the experimental period; complete nutritional analysis as provided by the manufacturer is found in Table 1. The rats' condition was observed daily, and experiments began after a 7-day acclimatization period. The experiments were conducted in strict accordance with the guidelines for the protection of experimental animals and were approved by the Animal Ethics Committee of Jining Medical College (Approval No.: 2021-DW-ZR-052). The rats were randomly divided into three groups (random number table; *n* = 8 per group): the Water group, the Diet Cola group, and the Cola group. Rats in the Water, Diet Cola, and Cola groups were provided with purified water, sugar-free cola, and sugar-sweetened cola, respectively, as their sole drinking source. The intervention lasted for 8 weeks.

**Table 1:** Complete nutritional analysis as provided by the manufacturer.

**Category**	**Specification**
Basal diet composition	Corn, wheat, soybean meal, soybean oil, limestone, fish meal, dicalcium phosphate, vitamins, and mineral premixes
**Nutrient levels**	
Moisture	≤ 100 g/kg
Crude protein	≥200 g/kg
Crude fat	≥40 g/kg
Crude fiber	≤ 50 g/kg
Crude ash	≤ 80 g/kg
Calcium (Ca)	10–18 g/kg
Phosphorus (P)	6–12 g/kg
Ca: P Ratio	1.2:1–1.7:1
Lysine	≥8.2 g/kg
Methionine + Cysteine	≥5.3 g/kg
Source	Jinan Pengyue Experimental Animal Breeding Co., Ltd., China

### 2.2 Blood routine measurements

After 8 weeks of intervention, rats were weighed, anesthetized with isoflurane, and the abdomen was dissected to expose the abdominal aorta for blood collection. A 2 ml whole blood sample was analyzed using a five-classification animal blood analyzer (Pukang PE-6700 Fully Automatic Blood Cell Analyzer) to determine white blood cell count, lymphocyte ratio, lymphocyte, granulocyte ratio, granulocyte, intermediate cell ratio, intermediate cell, total red blood cell count, hemoglobin, packed cell volume, mean erythrocyte volume, hemoglobin content, hemoglobin concentration, red cell distribution width, total blood platelet count, platelet distribution width, platelet hematocrit, and platelet-large cell ratio.

### 2.3 Blood biochemical measurements

Blood glucose was measured weekly from weeks 4–8 using a glucometer and blood glucose test strips, with blood collected from the tail vein. For other biochemical analyses, blood was allowed to clot for 30 min, then centrifuged at 12,000 r/min for 15 min at 4 °C. The resulting serum was analyzed using a BS-280 automatic biochemical analyzer (Mindray Medical Co., Ltd.) for total protein (TP), albumin (ALB), alanine aminotransferase (ALT), aspartate aminotransferase (AST), blood urea nitrogen (BUN), creatinine (Crea), creatine kinase (CK), triglycerides (TG), total cholesterol (T-CH), high-density lipoprotein (HDL), and low-density lipoprotein (LDL).

### 2.4 The calculation of body mass index (BMI), thymus index, and spleen index

Rats were weighed and measured for body length. BMI, thymus, and spleen indices were calculated according to the formula:


$$\begin{array}{l} \text{Thymus }(\text{Spleen) = }\frac{\text{Thymus }(\text{Spleen})\text{ mass }(\text{mg})}{\text{body mass }(\text{g})} \end{array}$$

$$\begin{array}{l} \text{ BMI = }\frac{\text{Body mass }(\text{g})}{\text{Body }{\text{length}}^{\text{2}}\;(\text{cm})} \end{array}$$

### 2.5 16S rRNA assay

Rat intestinal contents were collected and analyzed for intestinal microbiota diversity. The V3-V4 hypervariable region of the 16S rRNA gene was amplified and sequenced on an Illumina NovaSeq 6000 platform (Illumina, USA). The raw paired-end reads were processed using the DADA2 pipeline (version 1.26.0) within QIIME 2 to denoise, merge reads, and generate high-resolution ASVs (19, 20). Taxonomy was assigned against the SILVA 138 reference database (21). Subsequently, Alpha diversity, Beta diversity, community composition analysis, and LEfSe analysis were performed on the ASV table (22, 23). The network analysis is based on the relative abundance data of ASV. Spearman correlation analysis was employed (with the threshold set as |*R*| > 0.6 and *p* < 0.05). All the plots were created using the *R* packages ggraph_2.1.0 an and scatterpie_0.2.1.

### 2.6 Statistical analysis

GraphPad Prism software was used for data visualization and statistical analysis. After confirming normality (Shapiro–Wilk test) and homogeneity of variances (Brown–Forsythe test), One-way analysis of variance (ANOVA) was employed for multi-group comparisons. Statistically significant ANOVA results (*P* < 0.05) were followed by Tukey's *post-hoc* test for pairwise comparisons.

## 3 Results

### 3.1 Change in body weight, body length, BMI, thymus, and spleen indices in rats

**Figure 1:**
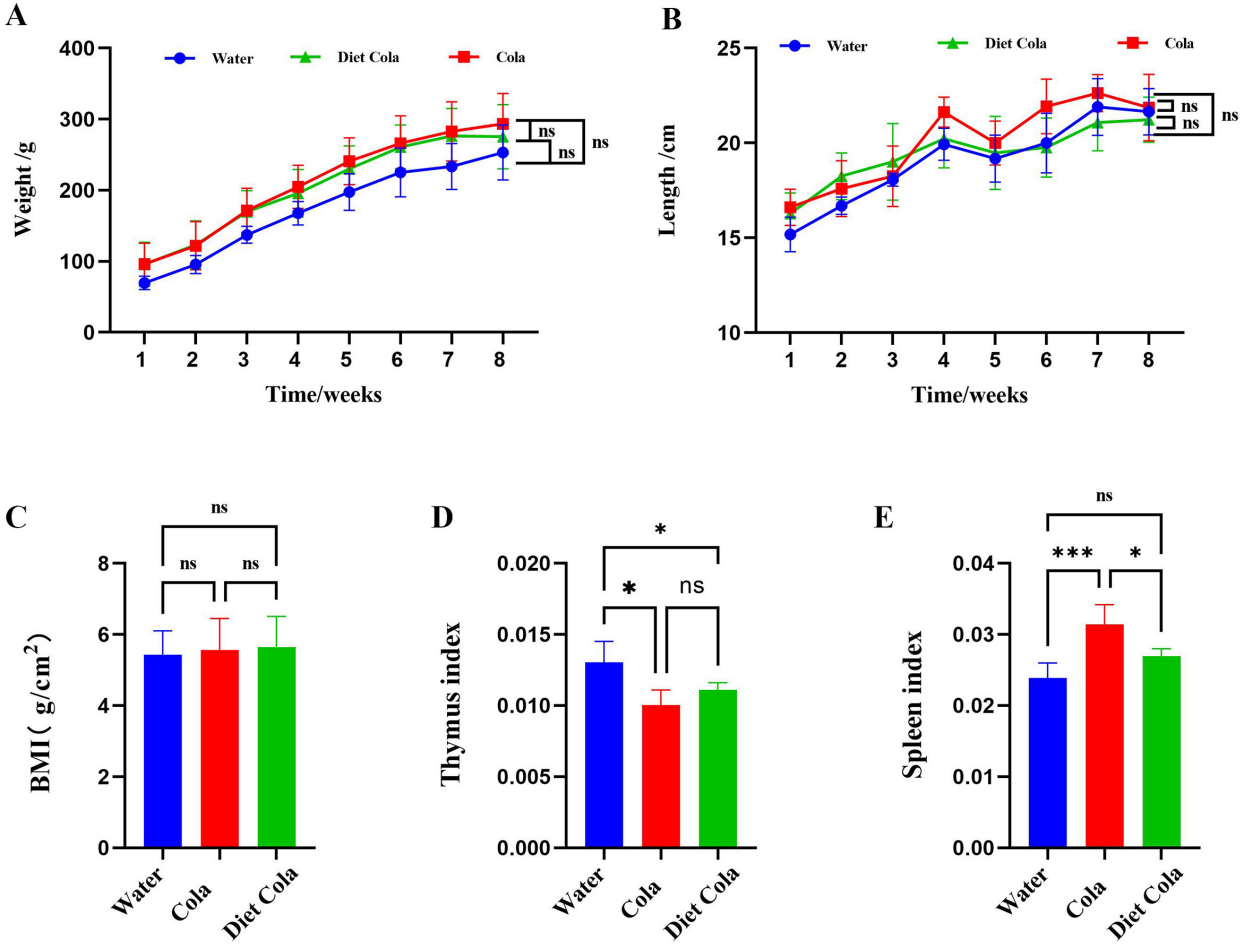
Change in **(A)** body weight; **(B)** body length; **(C)** BMI; **(D)** thymus; and **(E)** spleen indices in rats (ns *P* > 0.05, *P < 0.05, ***P < 0.001). The X-axis indicates the categorical treatment groups **(C–E)**.

The results showed no significant differences in body weight, body length, and BMI among the Water, Cola, and Diet Cola groups (Figures 1A–C). Compared to the Water group, rats in the Cola group exhibited a decrease in thymus index and an increase in spleen index. No significant differences in thymus and spleen indices were observed between the Diet Cola group and the Water group. The Cola group showed an increased spleen index compared to the Diet Cola group, while no significant difference was observed in the thymus index (Figures 1D, E).

### 3.2 Results of blood routine-related indexes in rats

**Figure 2:**
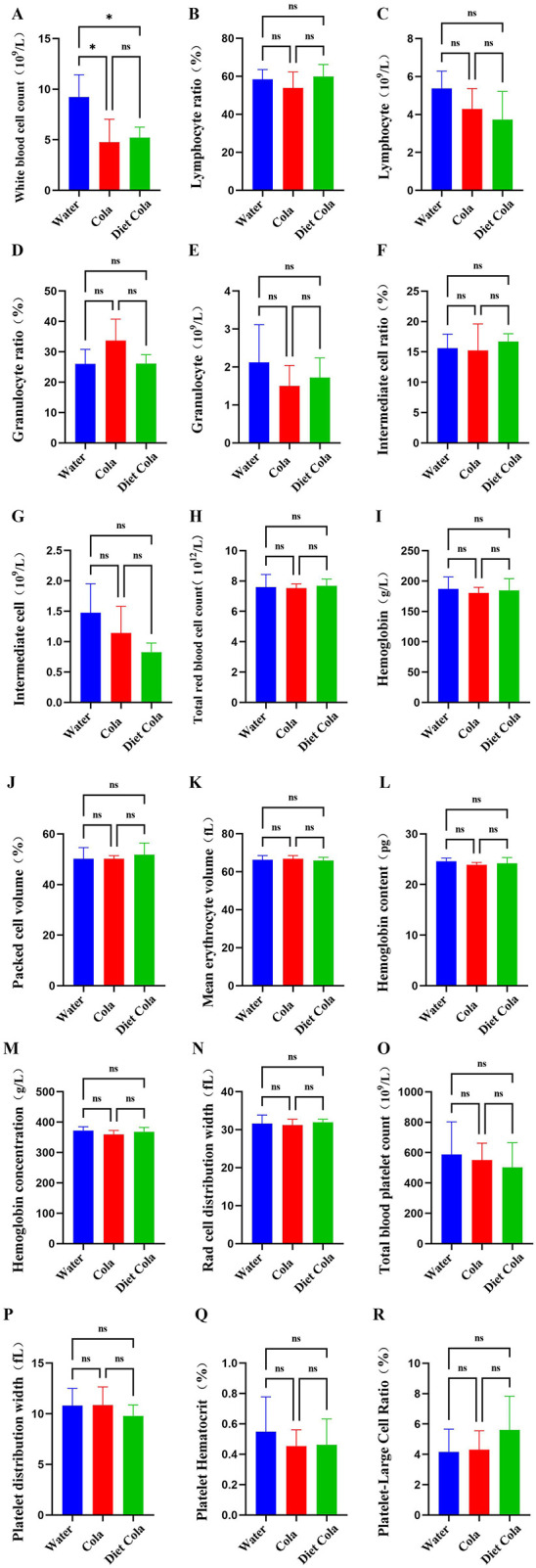
Blood routine of rats in each group: **(A)** white blood cell count; **(B)** lymphocyte ratio; **(C)** lymphocyte; **(D)** granulocyte ratio; **(E)** granulocyte; **(F)** intermediate cell ratio; **(G)** intermediate cell; **(H)** total red blood cell count; **(I)** hemoglobin; **(J)** packed cell volume; **(K)** mean erythrocyte volume; **(L)** hemoglobin content; **(M)** hemoglobin concentration; **(N)** red cell distribution width; **(O)** total blood platelet count; **(P)** platelet distribution width; **(Q)** platelet hematocrit; **(R)** platelet-large cell ratio (ns *P* > 0.05, *P < 0.05).

Compared to the Water group, the white blood cell count in whole blood was significantly reduced in both the Cola and Diet Cola groups. No significant differences were observed in lymphocyte ratio, lymphocyte, granulocyte ratio, granulocyte, intermediate cell ratio, intermediate cell, total red blood cell count, hemoglobin, packed cell volume, mean erythrocyte volume, hemoglobin content, hemoglobin concentration, red cell distribution width, total blood platelet count, platelet distribution width, platelet hematocrit, and platelet-large cell ratio. However, lymphocyte, granulocyte, and intermediate cell showed a downward trend (Figure 2).

### 3.3 Results of blood biochemistry-related indexes in rats

**Figure 3:**
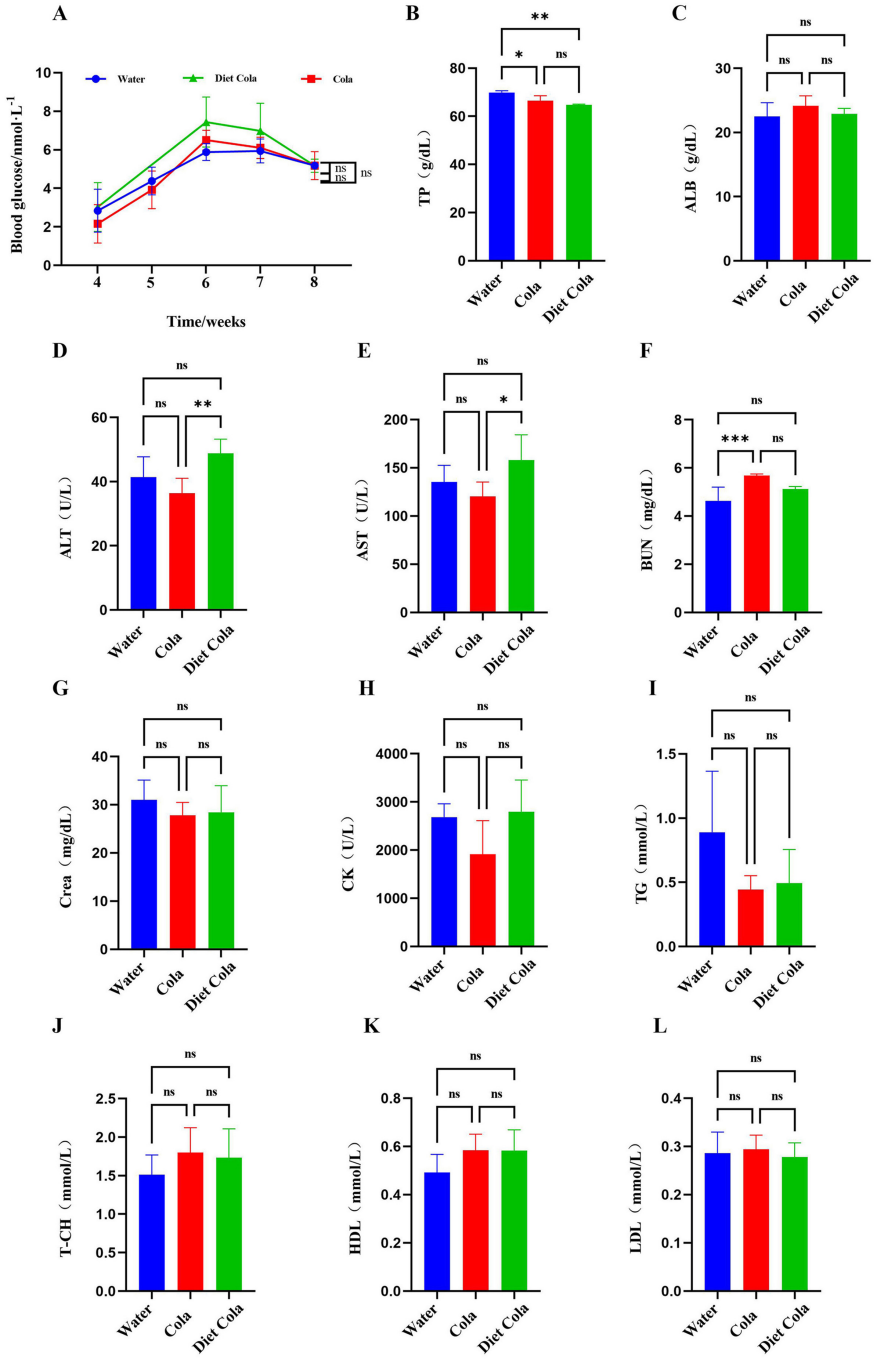
Blood biochemistry of rats in each group: **(A)** blood glucose; **(B)** TP; **(C)** ALB; **(D)** ALT; **(E)** AST; **(F)** BUN; **(G)** Crea; **(H)** CK; **(I)** TG; **(J)** T-CH; **(K)** HDL; **(L)** LDL (ns *P* > 0.05, *P < 0.05, **P < 0.01, ***P < 0.001).

The results showed no significant differences in blood glucose levels among the Water, Cola, and Diet Cola groups. Compared to the Water group, the Cola group exhibited decreased serum TP content and significantly increased BUN content. No significant differences were observed in the levels of ALB, ALT, AST, Crea, CK, TG, T-CH, HDL, and LDL. Compared to the Water group, in the Diet Cola group, there were no significant changes in other biochemical indicators except for a significant decrease in serum TP. Compared to the Cola group, the Diet Cola group showed increased levels of ALT and AST, with no significant differences in other blood biochemical indicators (Figure 3).

### 3.4 Analysis of sequencing results of intestinal flora

#### 3.4.1 Sequencing assessment of microbial 16S rRNA genes

**Figure 4:**
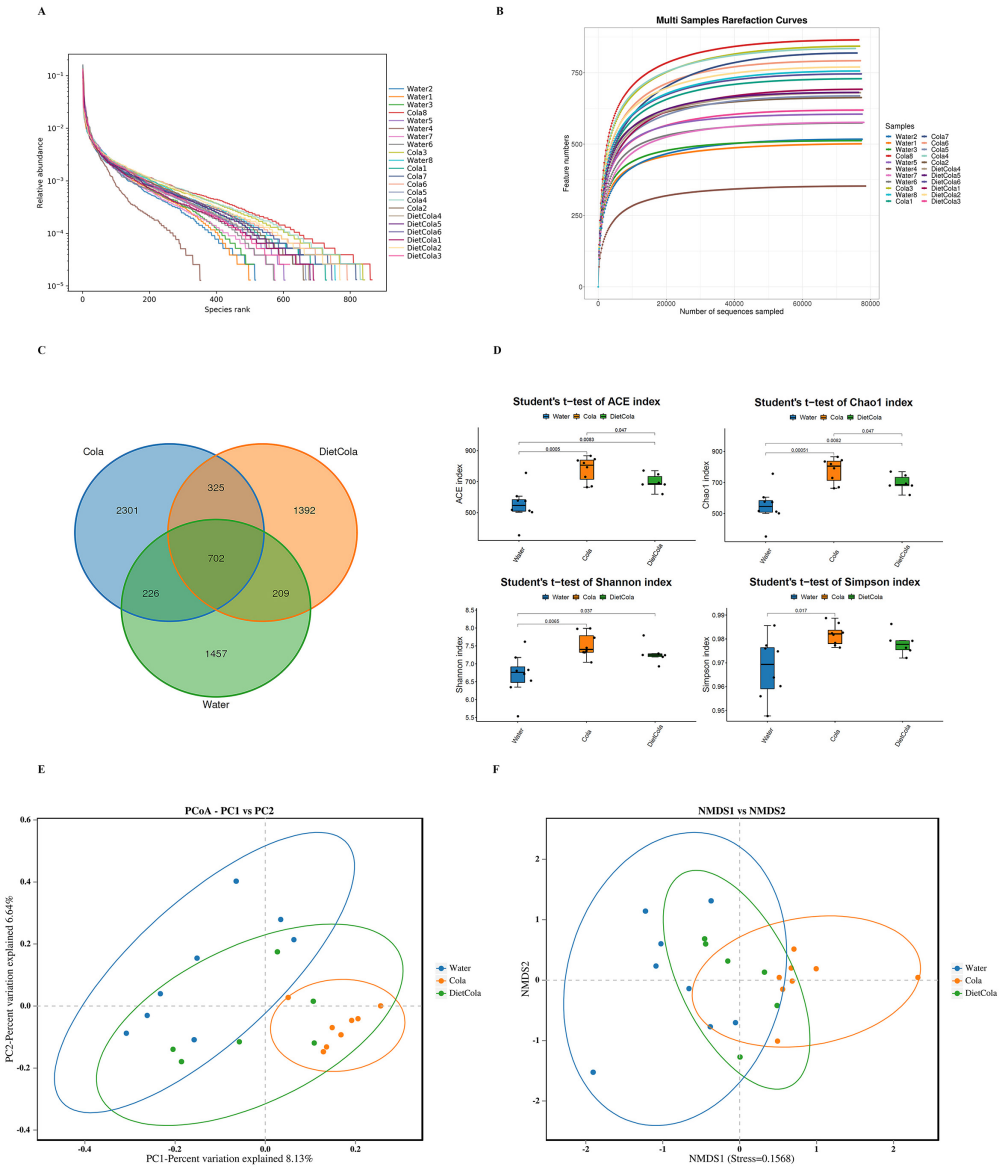
Results of 16Sr RNA sequencing analysis: **(A)** microbial 16S rRNA gene sequencing rank abundance curves; **(B)** dilution curves; **(C)** species characterization of rat intestinal flora Venn diagrams; **(D)** Alpha Diversity Index intergroup difference box line plots; **(E)** PCoA analysis plots; **(F)** NMDS analysis plots.

The Rank-Abundance Distribution curves for each experimental group were broad along the x-axis with the latter part of the curve approaching a horizontal asymptote suggesting that high diversity and evenness of species across samples (Figure 4A). The Rarefaction Curves for each group leveled off suggesting that the number of species detected did not increase substantially with increased sequencing depth and indicated sufficient coverage for the subsequent data analysis (Figure 4B).

#### 3.4.2 Overall sample microbial diversity analysis using OTUs

To determine the effects of cola consumption on the composition of rat intestinal microbiota, all samples were clustered into OTUs and annotated taxonomically. A Venn diagram illustrates the results (Figure 4C). A total of 702 OTUs overlapped among water, diet cola, and cola samples. The water group had 1,457 unique OTUs, the diet cola group had 1,392 unique OTUs, and the cola group had 2,301 unique OTUs. OTUs are perturbed by cola consumption (both sugar free and sugar sweetened) as compared to the water group. Interestingly, the cola group contained many more unique OTUs than the other two groups, indicating significant perturbation of the intestinal flora.

#### 3.4.3 Alpha diversity analysis of sample microbial community

Diversity of microbial communities was assessed using alpha diversity metrics. As presented in Figure 4D, both Cola and Diet Cola groups had higher ACE and Chao1 compared to Water group, indicating higher richness of intestinal microbiota species. Shannon and Simpson also increased indicating an increase in diversity. The ACE and Chao1 indices were significantly higher for Cola compared to Diet Cola, indicating a greater abundance of intestinal microbiota species in Cola.

#### 3.4.4 Beta diversity analysis of sample microbial communities

Beta diversity measures the differences in microbial community structures of disparate populations; the differences between samples were visualized using PCoA and NMDS. Cola group and Water group were well-separated suggesting that cola consumption impacted intestinal microbiota structure in rats. The Diet Cola group and Water group were less separated suggesting these groups had a more similar microbial community composition and abundance. Cola and Diet Cola groups were separated very minimally suggesting similar composition and species abundance of the microbial community (Figures 4E, F).

#### 3.4.5 Effects of cola and diet cola on the relative abundance of intestinal microbiota species in rats

**Figure 5:**
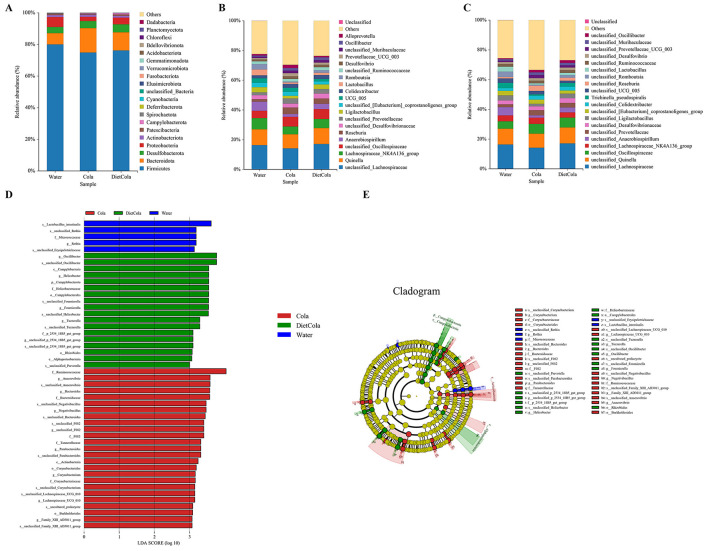
Histograms of species distribution and LEfSe multilevel discriminant analysis: **(A)** phylum level; **(B)** genus level; **(C)** species level; **(D)** histogram of LDA value distribution; **(E)** evolutionary branching diagram.

Based on taxonomic annotation, the top 20 most abundant taxa at the phylum level were selected to generate relative abundance bar charts for each group. As shown in Figure 5A, the dominant phyla in rat intestines were *Firmicutes, Bacteroidota, Desulfobacterota*, and *Proteobacteria*. Differences in phylum-level composition were observed among the three groups. The Cola and Diet Cola groups showed decreased relative abundance of *Firmicutes* and *Proteobacteria*, as well as the *Firmicutes/Bacteroidetes* ratio, and increased relative abundance of *Bacteroidota* and *Desulfobacterota* compared to the Water group. The Cola group exhibited lower relative abundance of *Firmicutes* and *Proteobacteria*, and higher relative abundance of *Bacteroidota* compared to the Diet Cola group.

The study revealed that the genera showing significant alterations were predominantly from the *Firmicutes* phylum. The Cola group exhibited a lower relative abundance of *unclassified_Lachnospiraceae, Quinella, Lachnospiraceae_NK4A136_group, Ligilactobacillus*, and *Lactobacillus* compared to Water group. Compared to the water group, the Diet Cola group showed no significant changes in the abundance of *unclassified_Lachnospiraceae, Quinella*, and *Ligilactobacillus*. The abundances of *Lachnospiraceae_NK4A136_group* and *Lactobacillus* decreased to some extent, but the magnitude of decrease was smaller than that in the Cola group (Figure 5B).

At the species level, the trends of microbial changes were relatively consistent with those observed at the genus level. The Cola group exhibited a lower relative abundance of *unclassified_Lachnospiraceae, unclassified_Quinella, unclassified_Ligilactobacillus*, and *unclassified_Lactobacillus* compared to Water group. Compared to the water group, the Diet Cola group showed no significant changes in the abundance of *unclassified_Lachnospiraceae, unclassified_Quinella*, and *unclassified_Ligilactobacillus*. The abundances of *unclassified_ Lactobacillus* decreased to some extent, but the magnitude of decrease was smaller than that in the Cola group (Figure 5C).

#### 3.4.6 Intestinal flora LEfSe multilevel species difference discriminant analysis

LEfSe multilevel discriminant analysis (with an LDA score threshold of 3) was employed to identify characteristic microbial taxa in each group. As shown in Figures 5D, E, 5, 18, and 25 significantly distinct bacterial communities were detected in the Water group, Diet Cola group, and Cola group, respectively, indicating substantial alterations in the intestinal microbiota structure across the groups.

#### 3.4.7 Co-abundance networks

**Figure 6:**
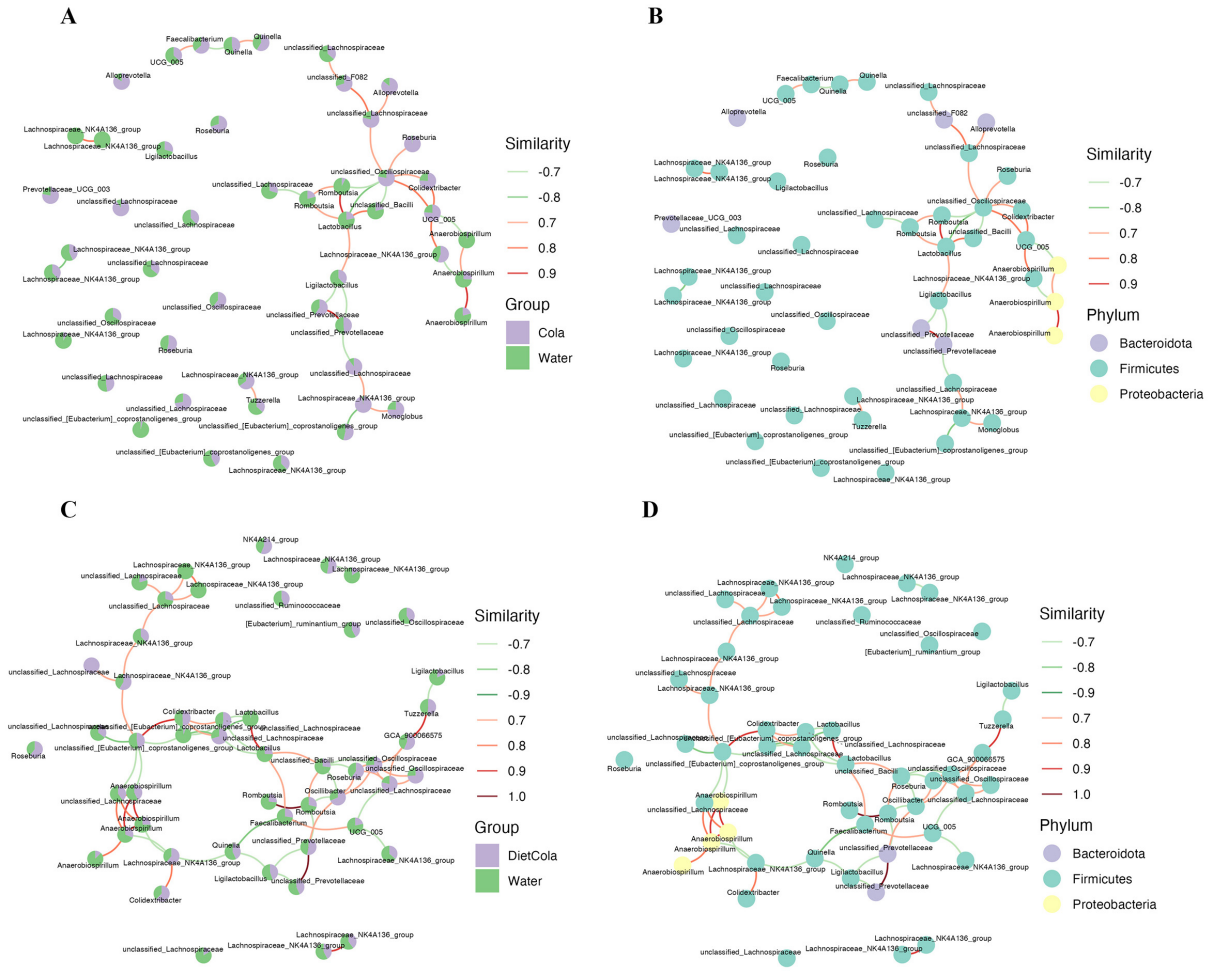
Co-abundance networks: **(A, C)** OTUs in the co-occurrence network were annotated in distinct groups; **(B, D)** OTUs in the co-occurrence network were annotated to different phyla.

The co-abundance network constructed from the Water and Cola groups revealed complicated connections, primarily among *Lactobacillus, Romboutsia, unclassified Prevotellaceae, Anaerobiospirillum, unclassified Oscillospiraceae*, and *UCG_005* (Figures 6A, B); the co-abundance network constructed from the Water and Diet Cola groups revealed complicated connections, primarily among *unclassified Prevotellaceae, Romboutsia, Lactobacillus, Anaerobiospirillum, unclassified_Lachnospiraceae*, and *Roseburia* (Figures 6C, D). Thus, these intestinal bacteria may exhibit either positive or negative associations, demonstrating symbiotic or competitive dynamics following treatment with Cola or Diet Cola.

#### 3.4.8 Analysis of the correlation between intestinal microbiota and phenotype

**Figure 7:**
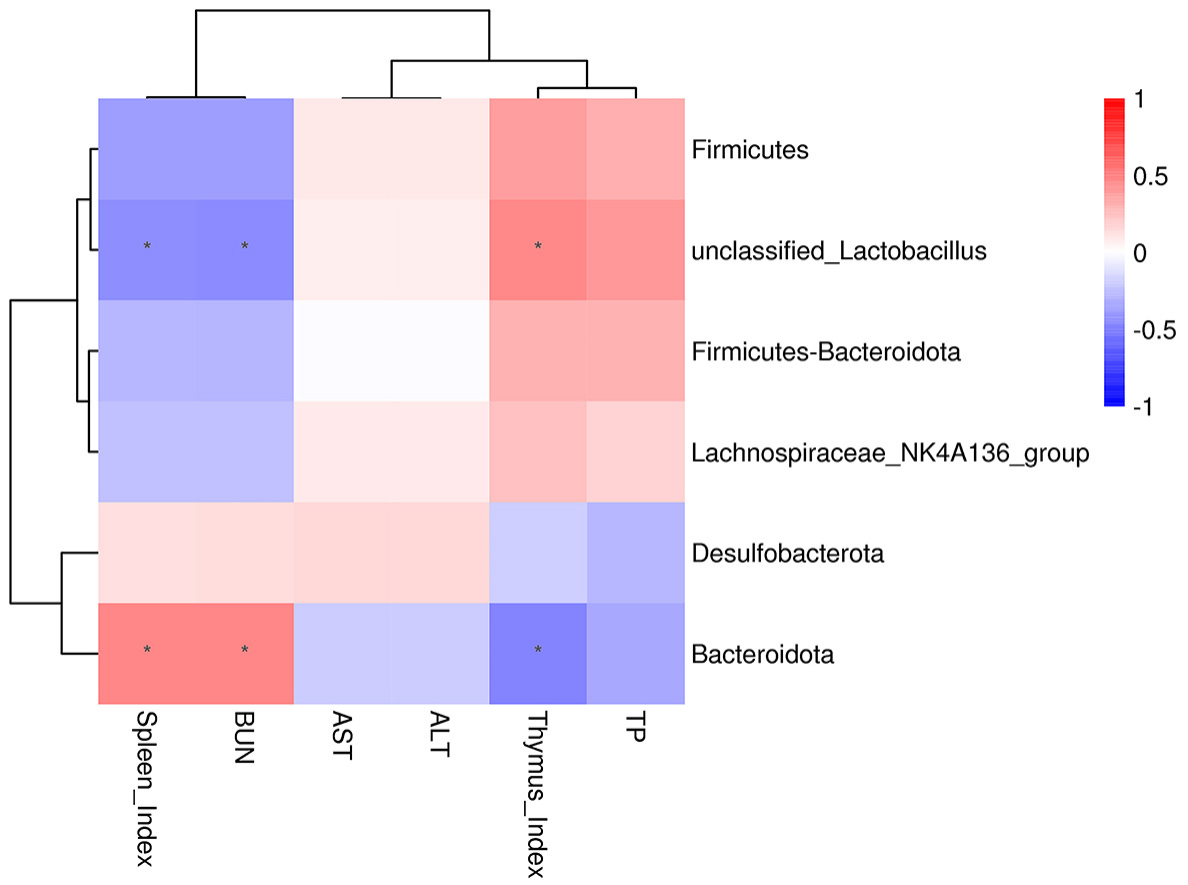
The correlation heatmap between intestinal microbiota and phenotype (*P < 0.05).

We used Pearson correlation analysis to assess the associations between gut microbiota composition and specific parameters, including the thymus-spleen index and serological markers. In Figure 7, *Bacteroidota* had a significant positive correlation with the spleen index and serum BUN content and negatively correlated with the thymus index (*P* < 0.05). Conversely, *unclassified Lactobacillus* had a negative relationship with both spleen index and serum BUN content and positively correlated with the thymus index (*P* < 0.05).

## 4 Discussion

Using a novel “cola as water substitute” model, this study shows that chronic ingestion of both sugar sweetened and sugar free cola disrupt systemic homeostasis in rats without overt changes in body weight, blood lipids or glucose metabolism. The key changes were immune suppression, substantial gut microbiota dysbiosis, and decreased total protein. Interestingly, sugar-sweetened cola caused renal stress and splenomegaly, while sugar free cola uniquely elevated transaminases.

Thymic atrophy is commonly associated with chronic low-grade inflammation, which impairs cellular immunity (24, 25). Concurrent splenomegaly suggests compensatory peripheral immune activation and hyperplasia, likely driven by systemic inflammation (26, 27). Our study found that the coexistence of decreased thymus index and increased spleen index indicates that cola consumption is associated with a certain degree of immune dysfunction. This finding is consistent with the observed leukocytopenia, both suggesting that cola consumption can lead to immune suppression and increased inflammation. Significant increase in BUN in the sugar sweetened cola group indicates renal toxic effect, which is alarming given kidneys function as a major metabolic clearance organ (28). Notably, the elevated ALT/AST levels and more pronounced decrease in TP specific to the diet cola group implicate hepatic implications of artificial sweeteners, corroborating epidemiological links between such sweeteners and metabolic liver disease (29–31). The above findings indicate that the health risks of cola are not only related to sugar.

In patients with active inflammatory bowel diseases (IBD), decreases in *Firmicutes* abundance and increases in *Bacteroidota* abundance have been observed (32). Cholesterol and oxidized cholesterol treatments in mice led to increased colitis and gut microbiota dysbiosis, with decreased relative abundance of short-chain fatty acid (SCFA)-producing bacteria (*Lachnospiraceae NK4A136 group and Blautia*) and increased abundance of potentially harmful bacteria (*Bacteroides*) (33). In our study, the Cola and Diet Cola groups resulted in a decrease in the relative abundance of *Firmicutes* and the *Firmicutes/Bacteroidota* ratio, and an increase in the relative abundance of *Bacteroidota* and *Desulfobacterota*.

Research has shown that the *unclassified Lachnospiraceae* and the *Lachnospiraceae NK4A136 group* exert a protective effect on metabolism and intestinal barrier through mechanisms such as lowering blood glucose and producing short-chain fatty acids (34–36); Other studies have shown that *Lactobacillus, Quinella* and *Ligilactobacillus*, as microorganisms with probiotic properties, play important roles in modulating the host microbiota and promoting health (37–39). The current analysis revealed that sugary cola reduced the abundance of *unclassified Lachnospiraceae, Quinella, Lachnospiraceae NK4A136 group, Ligilactobacillus*, and *Lactobacillus* within the phylum *Firmicutes*, while sugar-free cola only caused minor fluctuations in these bacterial communities, indicating a relatively limited perturbation effect. This indicates that diet cola has a lesser effect on the intestinal flora of rats compared to sugar-sweetened cola.

Research indicates that although *Lactobacillus* represents a minor component of the human colonic microbiota, its relative abundance frequently exhibits either a positive or negative correlation with various human diseases and chronic conditions, underscoring its relevance to human health (40). LEfSe and Co-occurrence network analysis further demonstrated complex interactions involving *Lactobacillus* in both sugar-sweetened cola and sugar-free cola. Correlation analysis revealed a significant association between *unclassified Lactobacillus* and the thoracic-spleen index. Thus, we speculate that *unclassified Lactobacillus* serves as a key component in the gut microbiota dysbiosis induced by replacing water with either regular or diet cola, and is closely linked to host physiological dysregulation.

Concurrently, we acknowledge the limitations of this study. Firstly, the experiment primarily assessed alterations in overall immune status and organ function through physiological and biochemical indicators in rats, without elucidating the specific mechanisms by which microbial changes influence host physiological functions. Secondly, the cola formulations tested are complex, hence it is difficult to ascertain whether an effect is due to component: the sweetener, phosphoric acid or caffeine etc.

To remedy these failings, several priorities for future work emerge. First, integrated multi-omics approaches, such as metabolomic or metatranscriptomic analysis, should be performed to reveal the active mechanism linking microbes with host physiology, followed by histopathological corroboration. Second, factorial studies, assessing the individual beverage components (artificial sweeteners, phosphoric acid, caffeine, etc.), must determine which are contributing more to the physiological and microbial parlance.

## 5 Conclusions

An innovative animal model where cola was substituted for water confirmed that chronic consumption of sugar-sweetened and sugar-free cola leads to significant systemic physiological dysfunction by changing gut microbiota composition. Sugar-sweetened cola tends to immunosuppression, greater renal burden, whereas sugar-free cola has a distinct tendency toward hepatotoxicity. Both of these findings lay-out that no cola is a safe option for hydration on a daily basis. Along with the relatively new finding that metabolic change, the current food safety assessment framework should integrate metabolic parameters and gut microbiota changes into the long-term safety evaluation criteria for artificial sweeteners. Our work not only reinforces the public health imperative to limit sugar-sweetened beverage intake but also calls for more stringent regulatory scrutiny of beverages containing non-nutritive sweeteners.

## Data Availability

The data presented in this study are publicly available. The data can be found here: https://www.ncbi.nlm.nih.gov/sra, PRJNA1413882.
